# *Gastrodia elata*, *Polygonatum sibiricum*, and *Poria cocos* as a Functional Food Formula: Cognitive Enhancement via Modulation of Hippocampal Neuroinflammation and Neuroprotection in Sleep-Restricted Mice

**DOI:** 10.3390/foods14071103

**Published:** 2025-03-22

**Authors:** Yiwen Zhang, Fang Chen, Xueyan Li, Yanfei Xu, Xinmin Liu, Muhammad Qasim Barkat, Muhammad Iqbal Choudhary, Qi Chang, Ning Jiang

**Affiliations:** 1 Research Center for Pharmacology and Toxicology, Institute of Medicinal Plant Development (IMPLAD), Chinese Academy of Medical Sciences and Peking Union Medical College, Beijing 100193, China; 2 Sino-Pakistan Center on Traditional Chinese Medicine, Hunan University of Medicine, Huaihua 418000, China; 3 Institute of Drug Discovery Technology, Ningbo University, Ningbo 315211, China; 4 H.E.J. Research Institute of Chemistry, International Center for Chemical and Biological Sciences, University of Karachi, Karachi 75270, Pakistan

**Keywords:** functional food, chronic sleep restriction, cognition, inflammation, neuroprotection, mice

## Abstract

*Gastrodia elata*, *Polygonatum sibiricum*, and *Poria cocos* are traditional Chinese herbs commonly used as both medicinal and food ingredients, traditionally believed to improve liver and kidney functions, replenish vital energy (qi) and blood, and mitigate stress-induced damage. These herbs are combined in the Compound Gastrodia elata Formula (CGEF), a functional food formulation. Amidst growing interest in functional foods, this study explores the cognitive-enhancing effects of CGEF, focusing on cognitive function improvement. Cognitive impairment was induced in ICR mice via chronic sleep restriction. Behavioral assessments including the Y-maze test, object recognition test, Morris water maze test, and Passive avoidance test, were conducted to evaluate CGEF’s effects. Serum levels of inflammatory markers and oxidative stress were quantified while in rat hippocampus tissue expressions of inflammatory, apoptotic, and neuroprotective-related protein markers were analyzed by Western blotting. Neurotransmitter concentrations in both the hippocampus and prefrontal cortex were determined by LC-MS/MS. CGEF significantly alleviated cognitive impairments across all behavioral tests. The underlying mechanisms likely involve a reduction in oxidative stress and peripheral inflammatory factors, and suppression of the TLR2/MyD88/NF-κB signaling cascade in the hippocampus, thereby mitigating neuroinflammation and neuronal apoptosis. Furthermore, CGEF modulated the PI3K/AKT/GSK3β signaling pathway, potentially contributing to neuronal integrity and synaptic plasticity maintenance. CGEF also restored neurotransmitter balance and regulated tryptophan metabolism, further alleviating cognitive deficits associated with sleep disruption. These findings suggest CGEF’s potential as a functional food for reversing cognitive impairments caused by chronic sleep restriction, primarily through its anti-inflammatory and neuroprotective effects.

## 1. Introduction

The rapid advancement of modern society has led to significant challenges in maintaining optimal sleep health. Factors such as a fast-paced lifestyle, prolonged screen exposure, and increasing work pressures have collectively contributed to a widespread decline in sleep quality [1,2]. Sleep disturbances not only compromise an individual’s quality of life satisfaction but are also strongly linked to cognitive impairments. Evidence suggests that chronic sleep deficiency or poor-quality sleep is associated with deficits in memory, attention, and executive function, potentially elevating the risk of developing dementia [3,4]. Cognitive impairments resulting from sleep deprivation diminish daily performance and work productivity, placing an increasing burden on healthcare systems and economic productivity [5]. Consequently, there is a growing demand for safe and effective treatments to counteract cognitive decline linked to sleep deprivation [6].

With increasing health awareness, the development of functional foods from food-medicine homologous herbs has gained significant attention, as these herbs are increasingly used for disease prevention and treatment [7,8]. The concept of “food therapy” (shizhi) dates back to the Tang Dynasty, as documented in the work “*Prescriptions Worth a Thousand Pieces of Gold for Emergencies* (Qianjin Yaofang)”, where it refers to utilizing food properties to regulate bodily functions for prevention, health maintenance, and disease treatment. Modern research supports the idea that diet can enhance cognitive function, and dietary interventions have been widely applied in wellness and healthcare [9,10]. *Gastrodia elata* Blume (Tianma) of the Orchidaceae family, *Polygonatum sibiricum* F. Delaroche (Huangjing) of the Asparagaceae family, and *Poria cocos* (Schw.) Wolf (Fuling) of the Polyporaceae family are commonly consumed in Chinese cuisine as food-medicine homologous substances. Traditionally, they are believed to replenish vital energy and blood, calm the liver, and soothe the mind, thereby enhancing cognitive functions. Pharmacological and clinical studies have demonstrated that these three herbs have long been recognized for their individual and synergistic effects on promoting cognitive function and enhancing sleep quality [11]. Modern pharmacology has shown that direct consumption of *Gastrodia elata* can improve cognitive function by reducing inflammation and apoptosis [12]. Among its active compounds, gastrodin and parishin exert significant neuroprotective effects, including improvements in sleep, cognitive function, and anxiety relief [13]. *Polygonatum sibiricum* enhances memory in aging rats by modulating the BDNF-TrkB signaling pathway [14] and the polysaccharides and saponins exhibit gut microbiota-regulating and antioxidative properties respectively, thereby improving cognitive function and slowing the aging process [15,16]. Similarly, *Poria cocos* has shown efficacy in alleviating cognitive impairments in Alzheimer’s disease models, with mechanisms attributed to the enhancement of acetylcholinesterase activity and the strengthening of antioxidant defenses within the brain [17,18]. Additionally, the polysaccharides of *Poria cocos*, which are its main active constituents, demonstrate significant anti-inflammatory effects [19]. The combination of these three herbs works synergistically to enhance therapeutic efficacy through mechanisms including the mitigation of inflammation, alleviation of oxidative burden, and neuroprotection.

This study evaluated a herbal functional food formula, Compound Gastrodia elata Formula (CGEF) consisting of *Gastrodia elata*, *Polygonatum sibiricum*, and *Poria cocos*. CGEF is derived from the classic traditional Chinese medicine formula “*Fuling Tianma Tang*”, which has shown promise in enhancing cognitive function. *Fuling Tianma Tang* was first documented in the “*Eye Classic*” (Yanke Jinghua Lu) and is traditionally used to treat symptoms caused by sleep deficiency and dampness, such as dizziness, insomnia, and mental fatigue [20]. Previous work in our laboratory demonstrated that CGEF synergistically enhances cognitive function in scopolamine-induced cognitive impairment mice, outperforming both individual ingredients and other combinations in the same model. As a result of its unique properties, this herbal functional food formula developed in this study has been patented in China, with the patent application number P0102024121165Z. To explore the potential mechanisms and therapeutic benefits of CGEF in alleviating cognitive damage resulting from chronic sleep deprivation, this study employed a mice model of cognitive impairment induced by such deprivation and finding the effect of CGEF in ameliorating cognitive deficiencies stemming from sleep restriction, focusing on aspects such as learning capacity, spatial memory, and declarative memory. Furthermore, its potential mechanisms of action through various aspects, including inflammation, oxidative stress, and synaptic plasticity were explored. This study aimed to develop and evaluate an efficacious herbal functional food approach for the prevention and management of cognitive impairments arising from sleep deprivation while providing robust experimental evidence to substantiate the scientific soundness and effectiveness of CGEF.

## 2. Materials and Methods

### 2.1. Preparation and Analysis of CGEF

**Figure 1:**
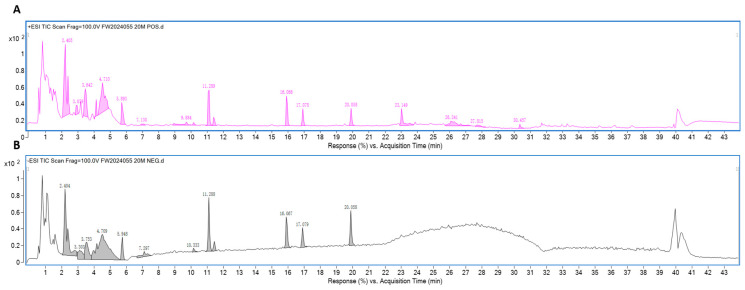
LCMS characterization of the constituents of CGEF. (**A**) The TIC chromatogram obtained in positive ion mode. (**B**) The TIC chromatogram obtained in negative ion mode.

The Compound Gastrodia elata Formula (CGEF) decoction was prepared by mixing *Gastrodia elata*, *Polygonatum sibiricum*, and *Poria cocos* in a ratio of 1:1:1. This mixture underwent three rounds of aqueous extraction using distilled water. The resulting extract was filtered through a 100-mesh sieve and subsequently concentrated to a final concentration of 1 g/mL. Chromatographic separation was executed on a Restek Ultra Aqueous C18 column (100 mm × 2.1 mm, 3 μm) (Restek, Centre County, PA, USA). Mass spectrometry analysis was conducted using an ESI source in both positive and negative ionization modes. Compound identification was by PCDL, SIRIUS, and phytochemical databases. A total of 28 characteristic peaks corresponding to the three herbs were identified, as shown in Figure 1. The analysis revealed the presence of active components including gastrodin, parishin, and p-hydroxybenzyl alcohol from *Gastrodia elata*, as well as various saponins, monosaccharides, and polysaccharides from *Polygonatum sibiricum* and *Poria cocos*. Detailed information on the identified compounds is provided in the Supplementary Materials.

### 2.2. Animals and Experimental Groups

A total of 144 SPF-grade male ICR mice aged 4–6 weeks were obtained from Charles River Laboratories in Beijing, China, under the qualification number SCXK(Jing) 2023-0008. All experiments were approved by the Committee for the Care and Use of Laboratory Animals at the Institute of Medicinal Plant Development (SLXD-20230409021). Animals were housed in a controlled environment with free access to standard feed and water. The house was maintained at a temperature of 23 ± 2 °C, a relative humidity of 55% ± 10%, and a 12 h light/dark cycle.

**Figure 2:**
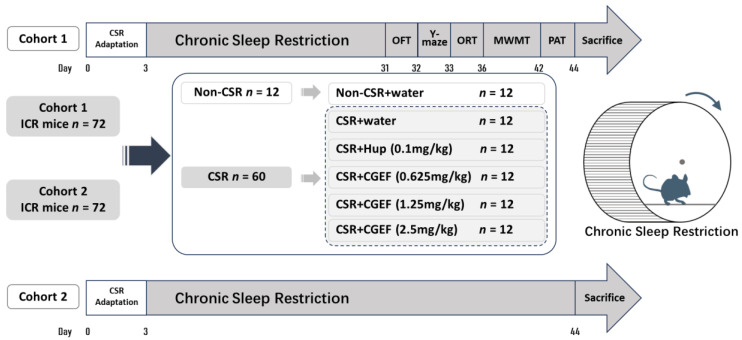
Diagram illustrating the experimental procedure. Note: OFT: open field test; ORT: object recognition test; MWMT: Morris water maze test; PAT: Passive avoidance test.

The experiment was conducted in two batches of 72 mice each. Following a one-week acclimatization period, mice in each batch were randomly assigned to one of six groups (*n* = 12 per group): the control group, received vehicle (distilled water); the chronic sleep restriction (CSR) group, received vehicle (distilled water); the positive control group, received Huperzine A at a dose of 0.1 mg/kg, and three groups receiving CGEF at low, medium, and high doses of 0.625 g/kg, 1.25 g/kg, and 2.5 g/kg, respectively. All groups except the control group underwent daily sleep restriction for a duration of four weeks. All treatments were administered via oral gavage at a volume of 10 mL/kg. The first batch of animals was examined for behavioral testing after four weeks of continuous sleep restriction and treatment. The second batch was subjected to the same duration of sleep restriction and treatments without behavioral testing and was subsequently sacrificed for tissue collection. The schematic representation of the experimental design is shown in Figure 2.

### 2.3. Chronic Sleep Restriction (CSR)

Chronic sleep restriction (CSR) was induced using a rotating drum apparatus based on previously described protocols with minor modifications [21]. Mice were acclimatized to the apparatus for three days prior to the start of the experiment. This involved gradual exposure to the rotating drum for 2, 4, and 6 h per day, respectively. During the CSR period, mice were placed in the rotating drum. The drum rotated at 1rpm for 1 min, followed by 2 min of rest. After each day’s behavioral testing, the animals were returned to the apparatus.

### 2.4. Behavioral Tests

#### 2.4.1. Open Field Test (OFT)

A circular black cylindrical testing chamber illuminated at 100 lux was used for the test. Animal movement was continuously monitored and recorded using a computerized tracking system. Each animal was gently placed in the testing chamber and allowed to acclimatize for 3 min to adjust before the test commenced. The spontaneous activity of the animals was recorded for 10 min.

#### 2.4.2. Y-Maze Test

The Y-maze apparatus was consisted of three equally spaced arms radiating from the central point. Mice were placed in the center of the maze and allowed to explore freely for 8 min. Their movements were tracked and recorded using ANY-maze software. Following each test, the maze was cleaned with 10% ethanol to eliminate any residual odors. The percentage of spontaneous alternation was calculated as: [(the number of successive triplets)/(the total number of arm entries − 2)] × 100%.

#### 2.4.3. Object Recognition Test

This test was performed as previously described [22]. Before testing, animals were given 10 min per day to freely explore and adapt to the experimental testing chamber. In the testing phase, mice were placed in the chamber with two identical objects positioned symmetrically on one side. They were allowed to explore for 5 min. In the novel object recognition test (NORT), after a 30 min interval, one familiar object was replaced with a new, unfamiliar object, while in the object location recognition experiment (OLRT), one familiar object was moved to the opposite side. To ensure that location preferences did not influence the results, the positions of the familiar and new objects or locations were balanced. The discrimination index (DI) was calculated which is the time spent exploring novel objects or locations divided by the total time spent exploring all objects.

#### 2.4.4. Morris Water Maze Test (MWMT)

The Morris water maze consisted of a circular pool filled with water made opaque by mixing black, non-toxic ink. A black platform submerged 1 cm below the water surface and placed in the center of the first quadrant. The spatial arrangement of the laboratory was kept unchanged throughout the experiment to provide spatial cues. Each animal was tested twice daily, with two entry points selected in sequence. Each trial in the positioning navigation training lasted for 90 s, and a trial was deemed successful if the animal located the platform and remained on it for at least 2 s. Following the completion of training, a spatial probe test was conducted 24 h later. The platform was removed from the pool and mice were allowed to swim freely for 60 s. The animals’ movement data were automatically recorded using a video tracking system.

#### 2.4.5. Passive Avoidance Test (PAT)

Each mouse was placed in a light chamber with its back facing the door to the dark chamber. After 3 min of free exploration to adapt to the apparatus, the floor grid of the dark chamber was electrified, and animals received a foot shock if they entered. The acquisition phase lasted for 5 min. Twenty-four hours later, in the consolidation phase, mice were again placed in the light chamber, with the grid in the dark chamber immediately electrified. The number of errors and the latency to enter the dark chamber for a maximum of 300 s were recorded.

### 2.5. Biochemical Analysis

#### 2.5.1. Preparation of Serum and Brain Tissue Samples

Following the completion of the behavioral tests, blood samples were collected from all mice via retro-orbital bleeding. Blood samples were stored at 4 °C overnight, then centrifuged at 3500 rpm for 20 min, and serum was collected and stored at −80 °C. After sacrificed by cervical dislocation, the brains were rapidly removed and the hippocampi and the prefrontal cortex were meticulously dissected on ice. Tissue samples were kept at −80°C for later analysis.

#### 2.5.2. Determination of Biochemical Parameters

Serum levels of malondialdehyde (MDA), hydrogen peroxide (H_2_O_2_), glutathione (GSH), glutathione disulfide (GSSG), superoxide dismutase (SOD) activity, and glutathione peroxidase (GSH-Px) activity were measured using commercially available assay kits from Jiancheng Biology, (Nanjing, China) according to the manufacturer’s instructions. Serum levels of tumor necrosis factor-alpha (TNF-α), interleukin-1 beta (IL-1β), and interleukin-6 (IL-6) were quantified using ELISA from Proteintech (Wuhan, China) following the manufacturer’s protocol.

#### 2.5.3. Western Blotting Analysis

Hippocampal tissue was homogenized in lysis buffer, and the protein-containing supernatant was extracted. Protein concentration was determined using a BCA protein assay kit (CWBIO, Taizhou, China). A standardized protein solution was then prepared, adjusting the concentration to 4 µg/µL with a mixture of protein lysis buffer and SDS-PAGE loading buffer. For protein separation, an SDS-PAGE system equipped with polyacrylamide gels ranging from 8–10% in concentration was employed. The separated proteins were transferred onto nitrocellulose membranes for further analysis. Blocking of the membranes with 5% non-fat milk was done at room temperature for one and a half hours, followed by incubation with primary antibodies overnight at 4 °C (Acetyl-NF-κB, 1:1000, Abcam; AKT, 1:1000, Abcam; BAX, 1:1000, Abcam; BCL2, 1:1000, Abcam; Caspase-8, 1:1000, CST; CREB, 1:10,000, Abcam; GSK3β, 1:10,000, CST; HO-1, 1:1000, CST; IL-1β, 1:1000, ImmunoWay; IL-6, 1:1000, Abcam; MyD88, 1:1000, CST; NF-κB, 1:1000, CST; Nrf2, 1:2500, Proteintech; P-AKT, 1:1000, Abcam; P-CREB, 1:5000, Abcam; P-GSK3β, 1:1000, CST; P-PI3K, 1:1000, Abcam; Phospho-NF-κB, 1:1000, Abcam; PI3K, 1:1000, Abcam; PSD95, 1:5000, Abcam; SYN, 1:10,000, Abcam; TLR2, 1:1000, CST; TrkB, 1:5000, Abcam; β-actin, 1:10,000, Proteintech). These membranes were then incubated with horseradish peroxidase-conjugated secondary antibodies at room temperature. Protein bands were visualized using enhanced chemiluminescence (ECL) reagent and quantified using ImageJ software((Version 1.8.0).

#### 2.5.4. Neurotransmitter Detection

Neurotransmitter analysis follows the method described by Wang et al. with slight variations [23]. Tissue neurotransmitter levels were determined using ultra-fast liquid chromatography and a QTRAP 5500 mass spectrometer (AB SCIEX, Framingham, MA, USA). Metabolite separation was achieved by gradient washing on a RESTEK ultra-water C18 column.

### 2.6. Statistical Analysis

Data were analyzed using SPSS version 26.0, and graphical representations were done through GraphPad Prism 8.0 software. For normally distributed data, a one-way ANOVA was performed, followed by the Least Significant Difference (LSD) post-hoc test. Alternatively, for non-normally distributed data, the Kruskal–Wallis H test was used. The outlier detection was performed using SPSS boxplot analysis to ensure data accuracy and reliability. All data are presented as mean ± SEM, with statistical significance defined as *p* < 0.05.

## 3. Result

### 3.1. Effect of CGEF on Locomotor Activity in Mice with CSR

**Figure 3:**
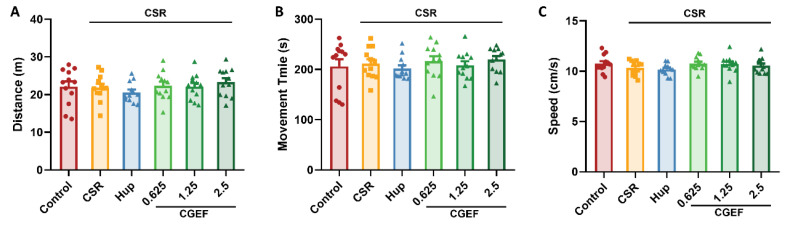
Effects of CGEF on locomotor activity in mice subjected to CSR. (**A**) Travel distance; (**B**) movement time; (**C**) movement speed. (*n* = 10–12, mean ± SEM).

No significant differences in distance traveled, movement time, or speed were observed among the experimental groups as shown in Figure 3 (all *p* > 0.05). These results indicate that neither CSR nor CGEF administration had any measurable impact on locomotor activity in mice.

### 3.2. Effect of CGEF on the Y-Maze Test

**Figure 4:**
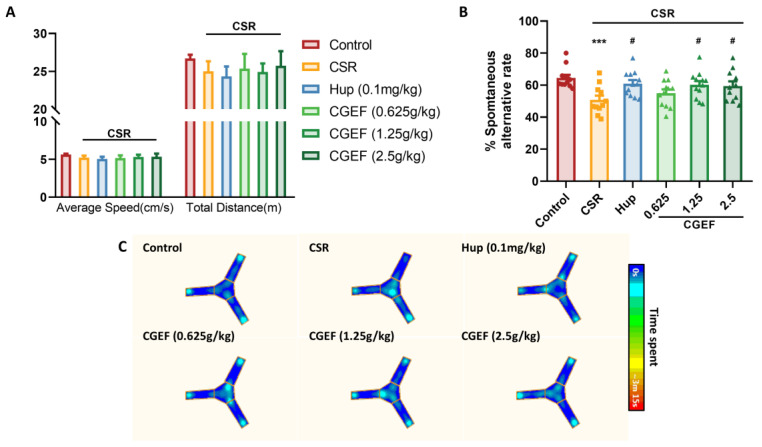
Effects of CGEF on Y-maze performance in CSR mice. (**A**) Locomotor speed and total distance; (**B**) spontaneous alternation rate; (**C**) representative movement heatmaps for each group. (*n* = 10–12, mean ± SEM), *** *p* < 0.001 vs. control; ^#^ *p* < 0.05 vs. CSR.

The Y-maze test results showed no statistically significant differences in locomotor activity among the experimental groups, indicating consistent movement levels across all groups (Figure 4A). However, the CSR group exhibited a significant reduction in the spontaneous alternation rate compared to the control group (*p <* 0.001), confirming that CSR induced short-term spatial memory impairment in mice (Figure 4B). Notably, both the positive control group and the CGEF-treated groups (1.25g/kg and 2.5g/kg) demonstrated significant improvements in the spontaneous alternation rate (*p <* 0.05), suggesting a protective effect against CSR-induced cognitive deficits. Representative heatmaps (Figure 4C) further illustrate that CSR mice exhibited repetitive entries into the same arms, whereas CGEF-treated groups showed enhanced exploratory behavior across all arms to varying degrees.

### 3.3. Effect of CGEF on Object Recognition Test

**Figure 5:**
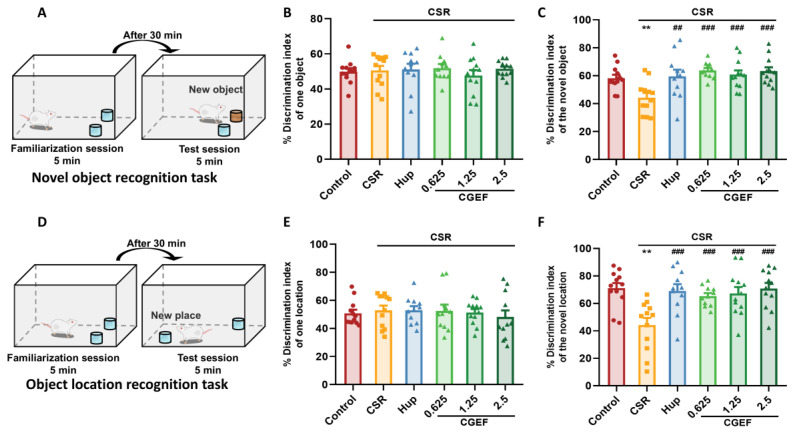
Effects of CGEF on object recognition performance in CSR mice. (**A**) Illustration of the novel object recognition test (NORT); (**B**,**C**) preference indices during the familiarization phase (**B**) and the test phase (**C**) of the NORT; (**D**) illustration of the object location recognition test (OLRT); (**E**,**F**) preference indices during the familiarization phase (**B**) and the test phase (**C**) of the OLRT. (*n* = 10–12, mean ± SEM). ** *p* < 0.01 vs. control; ^##^ *p* < 0.01, ^###^ *p* < 0.001 vs. CSR.

As shown in Figure 5, no significant differences in the preference index were observed among experimental groups during the familiarization phase, indicating a similar baseline object exploration. However, in both the NORT and OLRT, mice in the CSR group exhibited a significant decline in discrimination indices compared to the control group, suggesting impairments in novelty recognition and spatial memory. Statistical analysis confirmed these deficits, with significant differences detected in both tests (NORT: F_(5,64)_ = 5.28, *p <* 0.01; OLRT: F_(5,64)_ = 5.75, *p <* 0.01). Treatment with the positive control drug and all tested doses of CGEF significantly reversed the CSR-induced reduction in discrimination indices (*p <* 0.001 in both NORT and OLRT), indicating its potential to mitigate cognitive impairments associated with chronic sleep restriction.

### 3.4. Effect of CGEF on the Morris Water Maze Test

**Figure 6:**
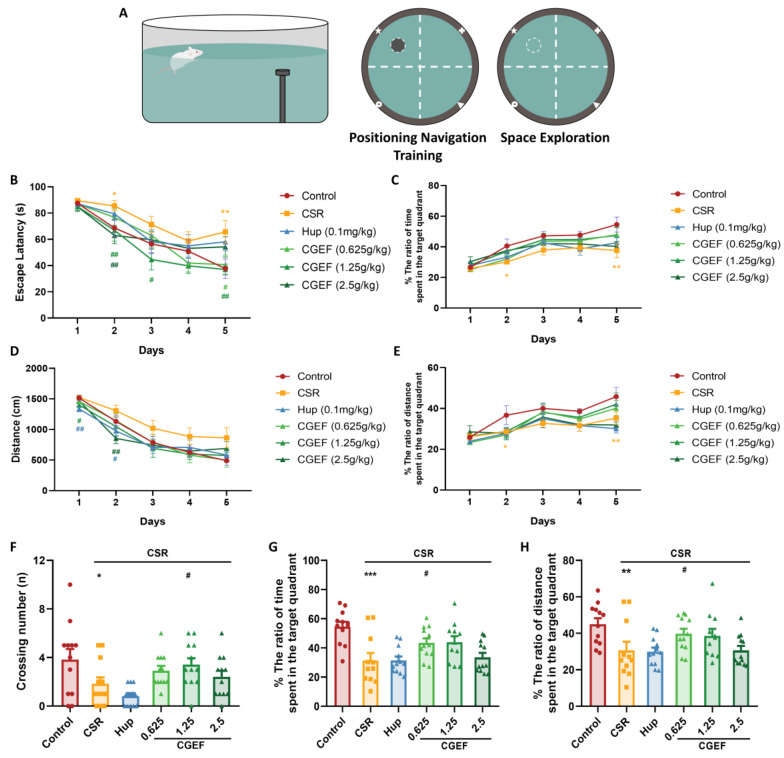
Effects of CGEF on Morris water maze Performance in CSR Mice. (**A**) Morris water maze schematic; (**B**,**C**) escape latency and target quadrant time ratio in navigation. (**D**,**E**) Total swimming distance and target quadrant distance ratio in navigation. (**F**–**H**) Platform crossings, target quadrant time/distance ratio in exploration. (*n* = 10–12, mean ± SEM). * *p* < 0.05, ** *p* < 0.01, *** *p* < 0.001 vs. control; ^#^ *p* < 0.05, ^##^ *p* < 0.011 vs. CSR.

During the positioning navigation training phase (Figure 6), the mice in the CSR group exhibited significantly prolonged escape latencies on Day 2 compared to the control group (*p* < 0.05). These deficits became more pronounced by Day 5, with latency significantly increased in the CSR group (F_(5,64)_ = 2.84, *p* < 0.01). CGEF administration mitigated these impairments in a dose-dependent manner: the low dose significantly reduced escape latency on Day 5 (*p* < 0.05), the medium dose improved performance on Days 2, 3, and 5 (*p* < 0.05), and the high dose was effective on Day 2 (*p* < 0.01). Regarding swimming distance, the positive drug group exhibited significant reductions on Days 1 and 2 (*p* < 0.05). CGEF groups also treatment led to notable decreases, with the medium dose on Day 1 (*p* < 0.05), and the high dose on Day 2 (*p* < 0.05). In the subsequent spatial probe test, CSR significantly reduced the number of platform crossings, as well as the percentage of time and distance spent in the target quadrant (*p* < 0.05, *p* < 0.001, and *p* < 0.01 respectively). Notably, the CGEF medium-dose group improved crossing times (*p* < 0.05); the low-dose group improved both target quadrant time and distance (*p* < 0.05).

### 3.5. Effect of CGEF on Passive Avoidance Test

**Figure 7:**
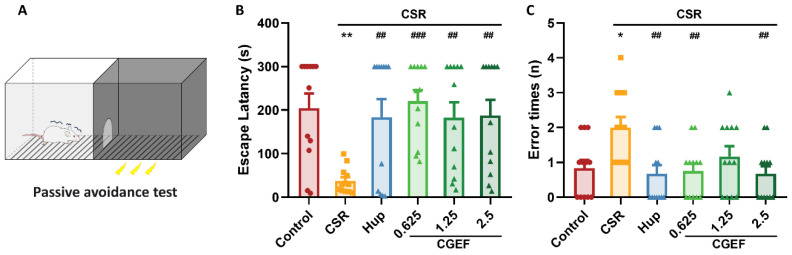
Effects of CGEF on passive avoidance performance in CSR mice. (**A**) Schematic diagram of the passive avoidance test. (**B**) Escape latency for entering the dark chamber. (**C**) Error times. (*n* = 10–12, mean ± SEM). * *p* < 0.05, ** *p* < 0.01 vs. control; ^##^ *p* < 0.01, ^###^ *p* < 0.001 vs. CSR.

The chronic sleep restriction (CSR) significantly reduced the latency to enter the dark chamber (*p* < 0.01) and increased the number of errors (*p* < 0.05) in the passive avoidance test compared to the control group. In contrast, treatment with the positive control drug and CGEF significantly prolonged the latency to enter the dark chamber (*p* < 0.01). Additionally, both the positive drug and CGEF at low and high doses significantly reduced the number of errors significantly (*p* < 0.01), suggesting a protective effect against CSR-induced cognitive impairment (Figure 7).

### 3.6. Effect of CGEF on Oxidative Stress Markers and Nrf2/HO-1 Pathway

**Figure 8:**
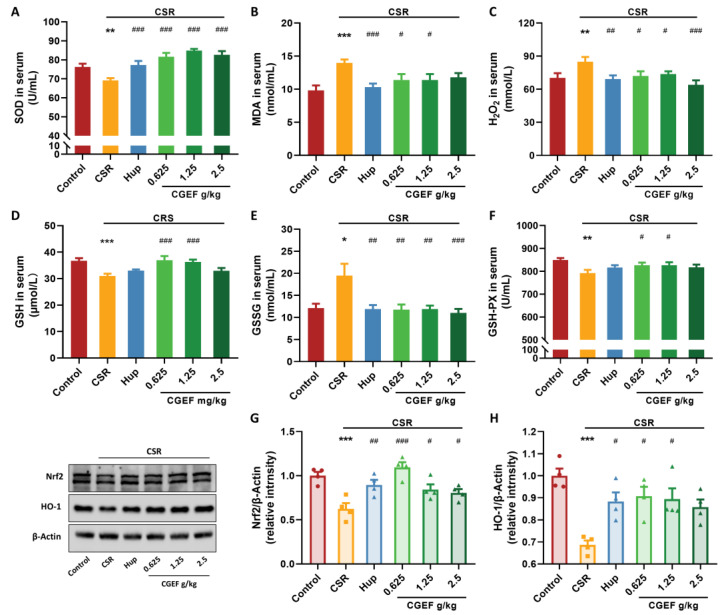
Effects of CGEF on oxidative stress-related biochemical indicators in mice with CSR. (**A**) SOD activity in serum; (**B**–**E**) the levels of MDA, H_2_O_2_, GSH, and GSSG in serum; (**F**) GSH-PX activity in serum. (**G**,**H**) Protein expression of Nrf2 and HO-1 in the hippocampus. (*n* = 9–10 in serum; *n* = 4 in hippocampus, mean ± SEM). * *p* < 0.05, ** *p* < 0.01, *** *p* < 0.001 vs. control; ^#^ *p* < 0.05, ^##^ *p* < 0.01, ^###^ *p* < 0.001 vs. CSR.

As shown in Figure 8, CSR induced significant oxidative stress in mice, as evidenced by altered serum oxidative markers. Specifically, CSR mice exhibited reduced SOD activity (F_(5,53)_ = 11.29, *p <* 0.01) and increased the levels of MDA, H_2_O_2_ (*p <* 0.001; F_(5,53)_ = 3.33, *p <* 0.01). Treatment with the positive control drug and low- or medium-dose CGEF significantly reversed these alterations (*p* < 0.05). Additionally, CSR led to a significant reduction in GSH levels and GSH-PX activity (F_(5.53)_ =5.72, *p <* 0.001; F_(5.53)_ = 2.49, *p <* 0.01), alongside an increase in GSSG (*p* < 0.05). Administration of low- and high-dose CGEF effectively inhibited excessive GSH oxidation (*p <* 0.05), with significant reductions in GSSG levels observed in the high-dose CGEF and Positive control groups (*p <* 0.01). In the hippocampus, CSR significantly decreased Nrf2 and HO-1 protein expression (F_(5,18)_ = 7.44, *p <* 0.001; *p <* 0.001). Treatment with CGEF and the positive control drug significantly restored Nrf2/HO-1 pathway activity (*p* < 0.05).

### 3.7. Effect of CGEF on Inflammatory Cytokine Levels in Serum and Hippocampus

**Figure 9:**
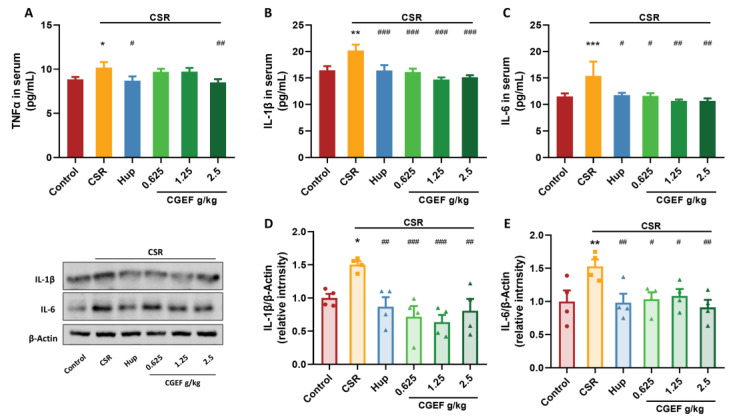
Effects of CGEF on inflammatory-related biochemical indicators in mice with CSR. (**A**–**C**) Levels of TNFα, IL-1β, and IL-6 in serum; (**D**–**E**) protein expression of IL-1β and IL-6 in the hippocampus. (*n* = 9–10 in serum, *n* = 4 in hippocampus, mean ± SEM). * *p* < 0.05, ** *p* < 0.01, *** *p* < 0.001 vs. control; ^#^ *p* < 0.05, ^##^ *p* < 0.01, ^###^ *p* < 0.001 vs. CSR.

Chronic sleep restriction (CSR) significantly increased serum levels of pro-inflammatory cytokines, including TNFα, IL-6, and IL-1β (F_(5,52)_ = 2.60, *p <* 0.05 for TNFα; F_(5,52)_ = 6.23, *p <* 0.01 for IL-1β; F_(5,52)_ = 2.45, *p <* 0.001 for IL-6). Treatment with the positive control drug and high-dose CGEF significantly suppressed these elevations (*p <* 0.05), while low- and medium-dose CGEF effectively reduced IL-1β levels (*p <* 0.001). In the hippocampus, CSR also led to a significant increase in IL-1β and IL-6 expression (F_(5,18)_ = 6.05, *p <* 0.05; F_(5,18)_ = 3.29, *p <* 0.01). The treatment with the positive control drug significantly reduced these inflammatory markers (*p <* 0.01). Notably, CGEF at all tested doses effectively reversed CSR-induced inflammatory cytokine activation in the hippocampus (*p <* 0.05), indicating its potential anti-inflammatory effects in mitigating CSR-related neuroinflammation (Figure 9).

### 3.8. Effect of CGEF on the TLR2/MyD88/NF-κB Pathway in the Hippocampus of Mice with CSR

**Figure 10:**
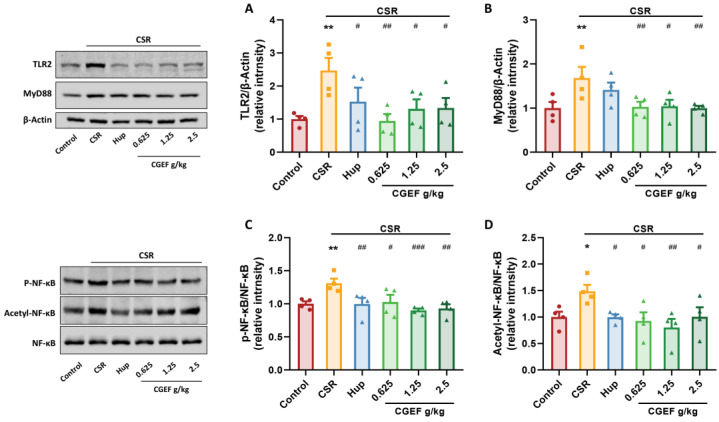
Effects of CGEF on the TLR2/MyD88/NF-κB pathway in mice with CSR. (**A**,**B**) Protein expression of TLR2 and MyD88; (**C**,**D**) protein expression of phosphorylated and acetylated NF-κB. (*n* = 4, mean ± SEM). * *p* < 0.05, ** *p* < 0.01vs. control; ^#^ *p* < 0.05, ^##^ *p* < 0.01, ^###^ *p* < 0.001 vs. CSR.

Our findings demonstrate that CSR significantly upregulated toll-like receptor 2 (TLR2) and myeloid differentiation factor 88 (MyD88) expression in mouse hippocampus (F_(5,18)_ = 3.35, *p <* 0.01; F_(5,18)_ = 3.30, *p <* 0.01). Additionally, CSR increased the phosphorylation and acetylation of nuclear factor-kappa B (NF-κB) (F_(5,18)_ = 4.13, *p <* 0.05; F_(5,18)_ = 2.88, *p <* 0.05). Hup treatment reduced TLR2, p-NF-κB, and acetyl-NF-κB levels (*p <* 0.05). Moreover, CGEF administration at all tested doses effectively inhibited activation of the TLR2/MyD88/NF-κB signaling pathway (*p <* 0.05) as shown in Figure 10.

### 3.9. Effect of CGEF on Apoptosis Protein Expression in the Hippocampus

**Figure 11:**
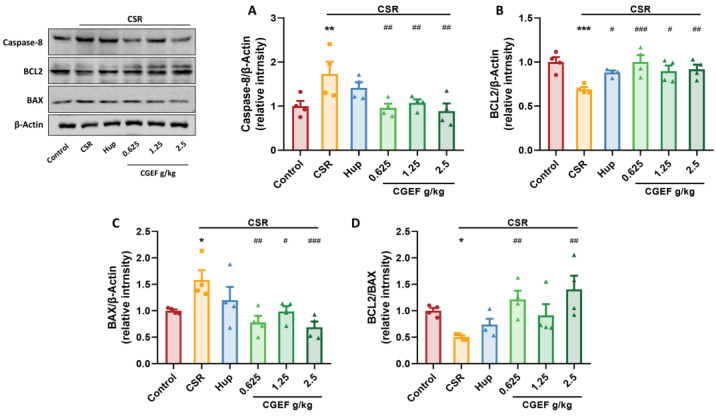
Effects of CGEF on apoptosis protein expression in the hippocampus of mice with CSR. (**A**–**C**) Protein expression of caspase-8, BCL2, and BAX; (**D**) ratio of BCL2 to BAX. (*n* = 4, mean ± SEM). * *p* < 0.05, ** *p* < 0.01, *** *p* < 0.001 vs. control; ^#^ *p* < 0.05, ^##^ *p* < 0.01, ^###^ *p* < 0.001 vs. CSR.

Chronic sleep restriction (CSR) significantly upregulated hippocampal expression of pro-apoptotic proteins caspase-8 and BAX (F_(5,18)_ = 2.87, *p <* 0.01; F_(5,18)_ = 4.58, *p <* 0.05), downregulating the anti-apoptotic protein BCL2 (F_(5,18)_ = 4.59, *p <* 0.001), leading to a disrupted BCL2/BAX balance (*p <* 0.05). Treatment with the positive control drug significantly increased BCL2 levels (*p <* 0.05). Administration of CGEF at all tested doses significantly reduced caspase-8 and BAX expression and increased BCL2 levels (all *p <* 0.05), and the abnormal BCL2/BAX ratio decline was significantly reversed in low- and high- dose CGEF groups (*p <* 0.01), indicating its potential to counteract CSR-induced apoptotic dysregulation in the hippocampus (Figure 11).

### 3.10. Effect of CGEF on PI3K/AKT/GSK3β and CREB Phosphorylation in the Hippocampus

**Figure 12:**
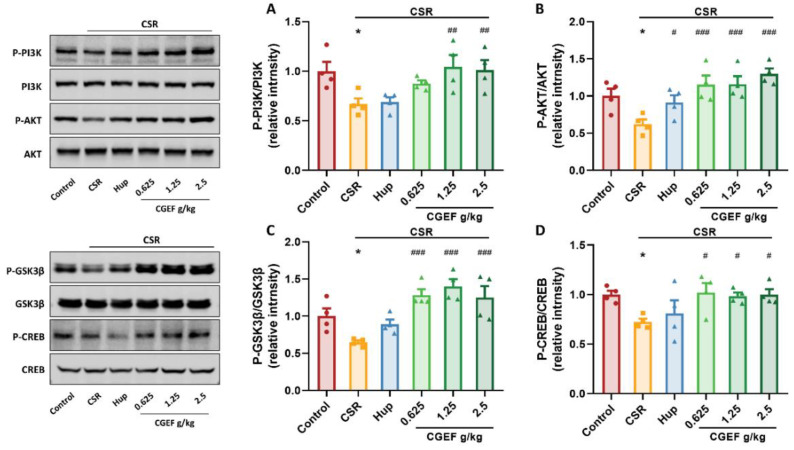
Effects of CGEF on the PI3K/AKT/GSK3β and CREB signaling pathways in the hippocampus of mice with CSR. (**A**–**D**) Phospho-PI3K/PI3K, phospho-AKT/AKT, phospho-GSK3β/GSK3β, and phospho-CREB/CREB levels. (*n* = 4, mean ± SEM). * *p* < 0.05vs. control; ^#^ *p* < 0.05, ^##^ *p* < 0.01, ^###^ *p* < 0.001 vs. CSR.

Our findings indicate that CSR significantly suppressed phosphorylation of key proteins in the PI3K/AKT/GSK3β signaling pathway in the hippocampus (F_(5,18)_ = 4.16, *p <* 0.05; F_(5,18)_ = 6.20; F_(5,18)_ = 8.76, *p <* 0.05), along with a reduction in cyclic AMP-responsive element-binding protein (CREB) phosphorylation (F_(5,18)_ = 2.77, *p <* 0.05). Treatment with the positive control drug significantly increased p-AKT levels (*p <* 0.05). While low-dose CGEF exhibited an increasing trend in p-PI3K, all doses of CGEF significantly restored phosphorylation levels of PI3K/AKT/GSK3β and CREB (*p <* 0.05), indicating its potential to counteract CSR-induced disruptions in these signaling pathways (Figure 12).

### 3.11. Effect of CGEF on Synaptic Plasticity and Neurotrophic Factor Expression in the Hippocampus

**Figure 13:**
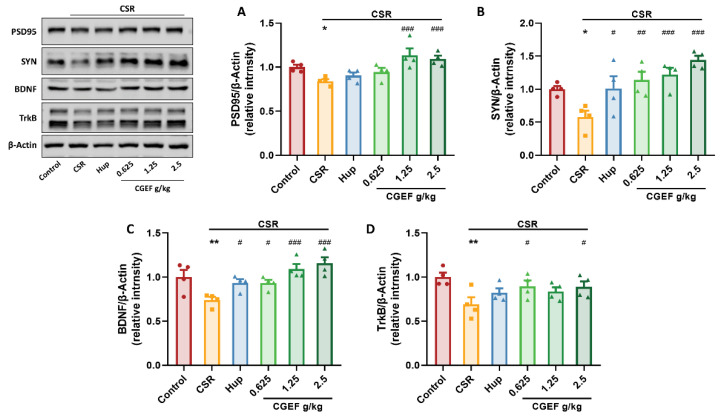
Effects of CGEF on the neuroprotective proteins in the hippocampus of mice with CSR. (**A**–**D**) Protein expression of PSD95, SYN, BDNF, and TrkB. (*n* = 4, mean ± SEM). * *p* < 0.05, ** *p* < 0.01 vs. control; ^#^ *p* < 0.05, ^##^ *p* < 0.01, ^###^ *p* < 0.001 vs. CSR.

Chronic sleep restriction significantly reduced the expression of synaptic proteins postsynaptic density protein-95 (PSD95) and synaptophysin (SYN) protein expression significantly decreased (F_(5,18)_ = 6.13, *p <* 0.05; F_(5,18)_ = 6.71, *p <* 0.05), as well as brain-derived neurotrophic factor (BDNF) and its receptor tropomyosin receptor kinase B (TrkB) (F_(5,18)_ = 6.69, *p <* 0.01; F_(5,18)_ = 2.83, *p <* 0.01). Treatment with the positive control drug significantly increased SYN and BDNF expression (*p <* 0.05). Administration of CGEF at all tested doses markedly enhanced SYN and BDNF levels (*p* < 0.05). Notably, high-dose CGEF markedly enhanced PSD95 expression (*p* < 0.001), while both low- and high-dose CGEF significantly upregulated TrkB expression (*p* < 0.05), indicating its potential to mitigate CSR-induced synaptic dysfunction and neurotrophic deficits (Figure 13).

### 3.12. Effect of CGEF on Neurotransmitter Levels in the Hippocampus and PFC

**Figure 14:**
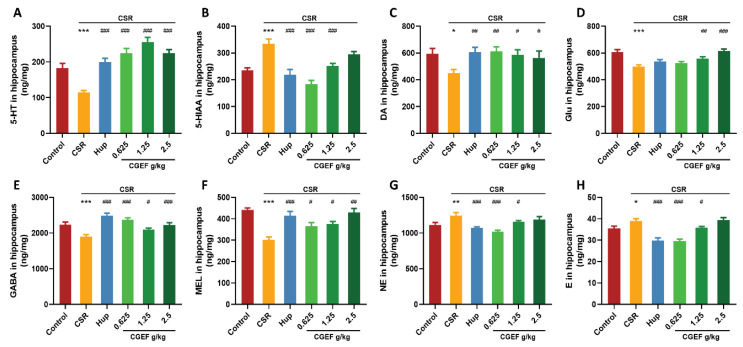
Effects of CGEF on neurotransmitter levels in the hippocampus of mice with CSR. (**A**) Levels of 5-HT; (**B**) levels of 5-HIAA; (**C**) levels of DA; (**D**) levels of Glu; (**E**) levels of GABA; (**F**) levels of MEL; (**G**) levels of NE; (**H**) levels of E. (*n* = 10–12, mean ± SEM). * *p* < 0.05, ** *p* < 0.01, *** *p* < 0.001 vs. control; ^#^ *p* < 0.05, ^##^ *p* < 0.01, ^###^ *p* < 0.001 vs. CSR.

The CSR significantly reduced hippocampal 5-Hydroxytryptamine (5-HT) levels compared to controls (F_(5,64)_ = 19.48, *p <* 0.001), while its metabolite 5-Hydroxyindoleacetic acid (5-HIAA) was significantly elevated (F_(5,64)_ = 14.45, *p <* 0.001). Additionally, CSR induced significant decreases in the hippocampal dopamine (DA), glutamate (Glu), gamma-aminobutyric acid (GABA), and melatonin (MEL) levels (F_(5,64)_ = 2.56, *p <* 0.05; F_(5,64)_ = 9.98, *p <* 0.001; F_(5,64)_ = 10.69, *p <* 0.001; F_(5,64)_ = 5.17, *p <* 0.001). In contrast, norepinephrine (NE) and epinephrine (E) levels were significantly increased in CSR mice (F_(5,64)_ = 7.01, *p <* 0.01; F_(5,64)_ = 19.12, *p <* 0.001). Treatment with the positive control drug significantly restored 5-HT and 5-HIAA levels (*p <* 0.001), increased DA, GABA, and MEL levels in the hippocampus (*p <* 0.01), and reduced NE and E levels (*p <* 0.01). Similarly, CGEF treatment at all doses effectively reversed CSR-induced abnormalities in 5-HT, DA, GABA, and MEL levels (*p <* 0.05). Low- and medium-doses significantly reduced 5-HIAA, NE, and E levels (*p* < 0.05), while medium- and high-dose CGEF significantly increased Glu levels (*p <* 0.01) as shown in Figure 14.

**Figure 15:**
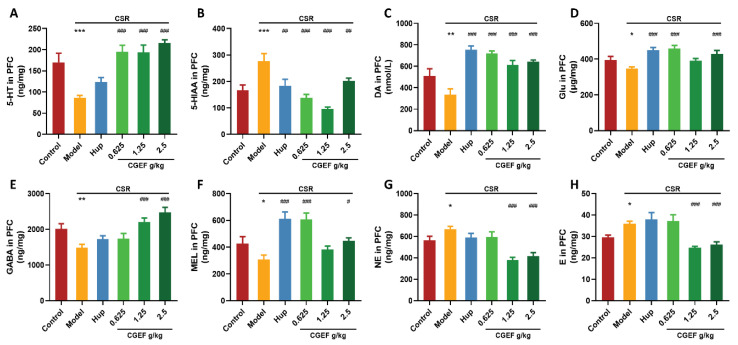
Effects of CGEF on neurotransmitter levels in the prefrontal cortex of mice with CSR. (**A**) Levels of 5-HT; (**B**) levels of 5-HIAA; (**C**) levels of DA; (**D**) levels of Glu; (**E**) levels of GABA; (**F**) levels of MEL; (**G**) levels of NE; (**H**) levels of E. (*n* = 10–12, mean ± SEM). * *p* < 0.05, ** *p* < 0.01, *** *p* < 0.001 vs. control; ^#^ *p* < 0.05, ^##^ *p* < 0.01, ^###^ *p* < 0.001 vs. CSR.

Neurotransmitter alterations in the prefrontal cortex (PFC) followed a similar trend. CSR led to a significant reduction in 5-HT (F_(5,64)_ = 11.84, *p <* 0.001) and increased 5-HIAA levels (F_(5,62)_ = 10.07, *p <* 0.001). Additionally, CSR reduced DA, Glu, GABA, and MEL levels (F_(5,64)_ = 12.89, *p <* 0.01; F_(5,64)_ = 6.60, *p <* 0.05; F_(5,64)_ = 8.24, *p <* 0.01; F_(5,64)_ = 9.46, *p <* 0.05), while increasing NE and E levels (F_(5,64)_ = 10.51, *p <* 0.05; F_(5,64)_ = 9.33, *p <* 0.05). Treatment with the positive control drug significantly decreased 5-HIAA (*p <* 0.01) and increased DA, Glu, and MEL levels (*p* < 0.05). CGEF administration at all doses reversed CSR-induced disruptions in 5-HT and 5-HIAA (*p* < 0.01) and significantly increased DA levels (*p* < 0.001). Furthermore, low- and high-dose CGEF enhanced Glu and MEL levels (*p <* 0.05), while medium- and high-dose CGEF increased GABA levels and reduced NE and E levels (*p <* 0.05) as shown in Figure 15.

### 3.13. Effect of CSR and CGEF on Tryptophan Metabolism in the Hippocampus and Prefrontal Cortex

**Figure 16:**
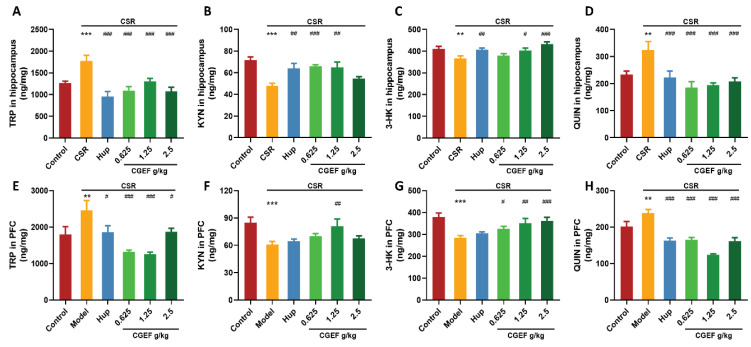
Effects of CGEF on tryptophan metabolism in the brain of mice with CSR. (**A**–**D**) Levels of TRP, KYN, 3-HK, and QUIN in the hippocampus; (**E**–**H**) levels of TRP, KYN, 3-HK, and QUIN in the PFC. (*n* = 9–10, mean ± SEM). ** *p* < 0.01, *** *p* < 0.001 vs. control; ^#^ *p* < 0.05, ^##^ *p* < 0.01, ^###^ *p* < 0.001 vs. CSR.

As shown in Figure 16, CSR significantly disrupted tryptophan (TRP) metabolism in both the hippocampus and PFC. The TRP levels were significantly increased in both regions (hippocampus: F_(5,65)_ = 9.76, *p <* 0.001; PFC: F_(5,63)_ = 7.46, *p <* 0.01), while kynurenine (KYN) and 3-hydroxykynurenine (3-HK) levels were markedly reduced (F_(5,65)_ = 6.62, *p <* 0.001 and F_(5,65)_ = 5.42, *p <* 0.01 in the hippocampus; F_(5,63)_ = 3.90, *p <* 0.001 and F_(5,64)_ = 6.06, *p <* 0.001 in PFC). Additionally, CSR significantly increased the levels of quinolinic acid (QUIN) (hippocampus: F_(5,65)_ = 6.37, *p <* 0.01; PFC: F_(5,64)_ = 20.13, *p <* 0.01), a neurotoxic metabolite associated with excitotoxicity and neuroinflammation. Treatment with the positive control drug effectively reversed TRP accumulation (*p <* 0.001), increased KYN and 3-HK levels (*p <* 0.01), and decreased QUIN levels (*p <* 0.001) in the hippocampus. In the PFC, positive control treatment prevented abnormal elevations of TRP and QUIN (*p <* 0.05). CGEF administration at all doses improved TRP metabolism to varying degrees. In the hippocampus, CGEF significantly reduced TRP and QUIN accumulation (*p <* 0.001), increased KYN levels at low and medium doses (*p <* 0.01), and elevated 3-HK levels at medium and high doses (*p <* 0.05). Similarly, in the PFC, CGEF reduced TRP and QUIN accumulation (*p <* 0.05), increased 3-HK levels (*p <* 0.05), and significantly enhanced KYN levels at the medium dose (*p <* 0.01).

## 4. Discussion

In this study, a series of cognitive behavioral tests were conducted to evaluate the efficacy of Compound Gastrodia elata Formula (CGEF) in mitigating cognitive impairments induced by chronic sleep restriction (CSR). Mechanistically, CGEF effectively attenuated oxidative stress and neuroinflammation in CSR-exposed mice by enhancing hippocampal antioxidant activity and suppressing inflammatory responses. The potential underlying mechanisms involve the inhibition of the TLR2/MYD88/NF-κB signaling pathway, leading to reduced neuroinflammation and apoptotic activity. Additionally, CGEF upregulated the PI3K/AKT/GSK3Β pathway, thereby restoring neuroprotective proteins and synaptic plasticity markers disrupted by CSR. Furthermore, CEGF contributes to maintaining neurotransmitter balance and preserving TRP metabolic homeostasis, highlighting its therapeutic potential in mitigating CSR-induced cognitive dysfunction.

Chronic sleep restriction (CSR) is an established model for replicating long-term sleep disturbances commonly observed in clinical settings. It is widely used to investigate the pathogenesis of sleep-related cognitive impairments and to identify potential cognitive-enhancing interventions [24]. In this study, five cognitive behavioral tests were employed to assess the neuroprotective effects of CGEF on spatial memory, working memory, and other cognitive domains. The Y-maze and object recognition tests (ORT), which are commonly used to evaluate short-term spatial memory and memory recall in rodents [25], demonstrated significant cognitive deficits in CSR-exposed mice, consistent with previous findings by Wang et al. [26]. However, CGEF treatment significantly improved the Y-maze alternation behavior and enhanced object discrimination and novel location recognition in the ORT, indicating its protective effect on short-term cognition and memory.

To further evaluate long-term cognition function, the Morris water maze test (MWMT), a widely used assessment of spatial learning and memory [27], was conducted. Notably, administration of a medium dose of CGEF significantly reduced escape latency during navigation training while increasing both the number of platform crossings and the time spent in the target quadrant during the spatial exploration phase. These findings indicate that CGEF effectively mitigates long-term spatial memory impairments induced by CSR. Additionally, the passive avoidance test (PAT), which assesses learning and memory based on conditioned reflexes and avoidance behavior [28], revealed that CGEF treatment significantly reduced escape latency and error frequency, suggesting improved learning acquisition and memory consolidation. Collectively, these findings demonstrate that CGEF reverses CSR-induced deficits in both short- and long-term memory, encompassing spatial and non-spatial memory impairments, while also enhancing learning and recall abilities. As a novel functional food formulation, CGEF holds significant potential for mitigating cognitive impairments associated with sleep deprivation.

Clinical studies have demonstrated that sleep deprivation exacerbates oxidative stress, a key factor closely associated with cognitive decline [29,30]. Consistent with these findings, the present study demonstrates that CSR significantly increases oxidative stress levels in mouse serum, corroborating previous research [31]. Moreover, both acute sleep deprivation and short-term chronic sleep disruption have been reported to significantly elevate oxidative stress in mice, reinforcing its pivotal role in sleep deprivation-induced cognitive impairment [32,33]. Importantly, the study identifies CGEF as an effective intervention for mitigating oxidative stress-induced damage. CGEF exerts its protective effects by modulating the activity of antioxidant enzymes including SOD and GSH-Px, while restoring the balance between antioxidants and oxidants such as GSH, MDA, H_2_O_2_, and GSSG. Furthermore, CGEF significantly upregulates the expression of Nrf2 / HO-1 proteins in the hippocampus. Nrf2 is a key player in cellular defense against oxidative stress, as its activation by reactive oxygen species (ROS), induces the expression of antioxidant proteins, particularly HO-1 [34,35]. Previous studies have demonstrated that activation of the NRF2/HO-1 pathway effectively reduces cognitive dysfunction in sleep deprivation stress models and other neurodegenerative conditions [36,37]. Therefore, CGEF may protect cognitive function by enhancing the brain’s antioxidant capacity through the NRF2/HO-1 pathway activation, thereby alleviating oxidative stress-induced neuronal damage.

Increasing evidence indicates that sleep deprivation significantly elevates pro-inflammatory cytokines, thereby triggering inflammatory processes that contribute to neuronal damage and cognitive dysfunction [38,39]. TLR2 (Toll-like receptor 2), is a key regulator of neuroinflammation in the central nervous system (CNS). When TLR2 is activated, it initiates a signaling cascade that promotes the expression of inflammatory mediators such as TNF-α, IL-1β, and IL-6, establishing a chronic pro-inflammatory environment [40,41]. The TLR2/MyD88/NF-κB pathway plays a central role in coordinating this inflammatory response. As a pattern recognition receptor, TLR2 detects both endogenous and exogenous stimuli, leading to the activation of downstream signaling molecules including MYD88 and NF-κB [42]. NF-κB, a key transcription factor, undergoes phosphorylation and translocates to the nuclear, where it derives the transcription of pro-inflammatory cytokines and inflammation-related proteins, thereby amplifying the inflammatory response [43]. This sustained neuroinflammation negatively impacts synaptic plasticity, neurogenesis, and apoptosis pathways, ultimately impairing cognitive functions such as learning and memory [44]. Our study demonstrates that CSR activates TLR2, leading to MYD88 recruitment and subsequently NF-κB activation. Notably, CGEF exerts potent inhibitory effects on the TLR2/MyD88/NF-κB signaling cascade, effectively reducing elevated inflammatory biomarker levels in both peripheral tissues and the CNS. This suppression of neuroinflammation may represent a key mechanism by which CGEF mitigates CSR-induced cognitive dysfunction.

Neuroinflammation is closely associated with neuronal apoptosis, a process that significantly contributes to cognitive impairment. Previous studies have demonstrated that sleep deprivation promotes neuronal death and cognitive deficits by regulating apoptosis-related proteins [45]. Caspase-8, a key effector enzyme in the apoptosis pathway, activates downstream caspase cascades, ultimately leading to neuronal death [46]. The pro-apoptotic protein such as BAX facilitates apoptosis by disrupting mitochondrial membrane integrity, whereas BCL-2 counteracts BAX activity, thereby inhibiting apoptosis and promoting cell survival. [47]. Our study demonstrates that CGEF treatment significantly enhances BCL-2 expression while suppressing the activation of caspase-8 and BAX, thereby reducing neuronal apoptosis in the hippocampal. These findings suggest that CGEF exerts neuroprotective effects against sleep deprivation-induced neuronal damage by modulating key apoptotic signaling pathways, highlighting its potential as a therapeutic intervention for cognitive impairment.

The PI3K/AKT/GSK3β pathway plays a fundamental role in regulating vital biological functions such as cellular survival, metabolism, and apoptosis, with its activation being crucial for neuroprotection and neuronal viability [48]. Prolonged external stressors can inhibit the phosphorylation of PI3K (phosphoinositide 3-kinase), leading to suppression of its downstream effector AKT (Protein Kinase B) and subsequent hyperactivation of GSK3β (glycogen synthase kinase 3β) via reduced Ser9 phosphorylation [49]. Consistent with our findings, previous studies have reported that CRS inhibits the PI3K/Akt pathway, resulting in hippocampal damage and cognitive deficits [50]. GSK3β, a key downstream target of this pathway, has a dual role in synaptic regulation. Moderate inhibition of GSK3β enhances neuroprotection by stabilizing PSD95, thereby improving synaptic signaling and plasticity [51]. However, excessive GSK3β activation contributes to neuroinflammation, oxidative stress, and the downregulation of synaptic proteins, ultimately impairing learning and memory [52,53]. Our findings indicate that CSR induces excessive GSK3β activation by suppressing the PI3K/Akt signaling, leading to neuronal dysfunction. Notably, CGEF treatment effectively restored GSK3β Ser9 phosphorylation, increased the expression of synaptic proteins PSD95 and SYN, and enhanced neuronal plasticity. These findings suggest that CGEF exerts neuroprotective effect against CSR-induced neuronal damage by modulating the PI3K/AKT/GSK3β signaling pathway.

Furthermore, CGEF has been shown to inhibit GSK3β activity, thereby facilitating the activation of cAMP-responsive element-binding protein (CREB) [54]. As a key transcription factor, CREB induces the expression of neuroprotective proteins including BDNF, which promotes synaptic strengthening and neuronal plasticity [55]. BDNF exerts its neuroprotective effects by binding to its high-affinity receptor, TrkB, initiating a signaling cascade that promotes neuronal survival and synaptic maintenance [56]. The activation of BDNF/TrkB signaling further stimulates the PI3K/AKT pathway, creating a positive feedback loop that enhances synaptic protein expression, such as PSD95 and SYN. These proteins are essential for neural development, synaptic plasticity, neurotransmission, and memory formation [57,58]. Research indicates that TrkB activation is instrumental in improving neuronal plasticity, which is crucial for maintaining neural integrity and cognitive function [59]. Collectively, these molecular mechanisms suggest that CGEF may protect against CSR-induced cognitive impairment by activating the PI3K/AKT/GSK3Β pathway and upregulating neuroprotective proteins, ultimately enhancing synaptic function and neuronal plasticity.

The CNS is highly sensitive to the detrimental effects of sleep deprivation, which can lead to complex alterations in neurotransmitter homeostasis [60]. Neurotransmitters are essential for signal transmission in the CNS, and imbalances in their levels can contribute to neuronal dysfunction and cognitive impairments [61]. Among these, serotonin (5-HT) plays a crucial role in cognitive processes, with reduced 5-HT levels commonly associated with neurodegenerative diseases such as Alzheimer’s disease and other cognitive disorders [62]. Our findings demonstrate that CSR significantly decreased 5-HT levels while increasing 5-HIAA (a 5-HT metabolite), suggesting impaired 5-HT synthesis coupled with accelerated metabolism. CGEF treatment effectively restored 5-HT levels and significantly reduced 5-HIAA, indicating that CGEF mitigated CSR-induced neurotransmission dysregulation and improved cognitive function. Furthermore, CGEF alleviated sleep deprivation-induced alterations in several other key neurotransmitter levels, including dopamine (DA), glutamate (Glu), gamma-aminobutyric acid (GABA), melatonin (MEL), norepinephrine (NE), and epinephrine (E). DA deficiency has been strongly linked to reduce cognitive flexibility [63], while, an imbalance between the primary excitatory neurotransmitter Glu and the inhibitory neurotransmitter GABA can weaken synaptic strength, leading to cognitive decline [64]. A decrease in MEL reflects disruptions in sleep rhythm regulation [65], and elevated NE and E levels indicate potential hyperactivation of the HPA axis due to prolonged sleep deprivation, resulting in excessive CNS activity and dysfunction associated with cognitive impairment [66,67]. Overall, these findings suggest that CGEF plays a critical role in restoring neurotransmitter homeostasis, thereby counteracting CSR-induced neurochemical dysregulation and cognitive deficits.

Tryptophan (TRP) is a critical precursor for 5-HT synthesis and a key component of the kynurenine (KYN) metabolic pathway, which accounts for approximately 95% of TRP metabolism. This pathway produces several metabolites including KYN, 3-hydroxykynurenine (3-HK), and quinolinic acid (QUIN) [68]. Previous studies have demonstrated that sleep deprivation leads to excessive 5-HT consumption and disruptions in TRP metabolism, particularly through the accumulation of neurotoxic TRP metabolites [69,70]. In our study, sleep deprivation resulted in significantly elevated TRP levels and a pronounced increase in QUIN, a key metabolite in the TRP-KYN pathway. QUIN is highly neurotoxic due to its ability to activate NMDA receptors, leading to excitotoxicity and contributing to cognitive impairments and various neurodegenerative diseases [71]. However, CGEF treatment effectively counteracted these metabolic alterations, suggesting that CGEF mitigates overactivation of the TRP-KYN pathway by inhibiting QUIN production. By restoring normal KYN metabolism, CGEF reduces the accumulation of neurotoxic metabolites, thereby protecting neurons from excitotoxic damage and potentially improving cognitive function. These findings highlight CGEF’s therapeutic potential in counteracting sleep deprivation-induced TRP metabolism dysregulation and its associated neurotoxic effects.

## 5. Conclusions

This study provides an in-depth investigation into the effects of Compound Gastrodia elata Formula (CGEF), a functional herbal food formula composed of *Gastrodia elata*, *Polygonatum sibiricum*, and *Poria cocos*, on cognitive impairment induced by chronic sleep restriction (CSR) in mice. The findings demonstrate that CGEF significantly mitigates CSR-induced cognitive dysfunction through multiple mechanisms. These include inhibition of the TLR2/MyD88/NF-κB pathway to suppress neuroinflammation and modulation of the PI3K/Akt/GSK3β pathway to maintain neuronal integrity and synaptic plasticity. Additionally, CGEF restores neurotransmitter homeostasis and regulates tryptophan metabolism, further mitigating cognitive deficits associated with sleep disruption. Overall, the multi-target effects of CGEF highlight its therapeutic potential in addressing cognitive impairments associated with sleep restriction, while also underscoring the broader applicability of food-medicine homologous products in modern scientific research. By elucidating the molecular pathways through which CSR disrupts cognitive function and how CGEF counteracts these effects, this study provides valuable insights into the development of functional food as potential interventions for cognitive impairments.

## Data Availability

The original contributions presented in this study are included in the article/Supplementary Materials. Further inquiries can be directed to the corresponding authors.

## Supplementary Materials

The following supporting information can be downloaded at: https://www.mdpi.com/article/10.3390/foods14071103/s1, Figure S1. Identification of compounds in CGEF by LCMS. (A) Total ion chromatogram (TIC) of CGEF in positive ion mode; (B) Total ion chromatogram (TIC) of CGEF in negative ion mode; (C) UV absorbance spectrum of CGEF at 254 nm; Table S1: Compound identification information of CGEF by LCMS.

## Abbreviations

The following abbreviations are used in this manuscript, listed in alphabetical order:

3-HK	3-Hydroxykynurenine
5-HIAA	5-Hydroxyindoleacetic acid
5-HT	5-Hydroxytryptamine (Serotonin)
BCL2	B-cell lymphoma 2
BAX	BCL2-associated X protein
BDNF	Brain-derived neurotrophic factor
CREB	cAMP-responsive element-binding protein
CSR	Chronic sleep restriction
DA	Dopamine
E	Epinephrine
GABA	Gamma-aminobutyric acid
Glu	Glutamate
HO-1	Heme oxygenase-1
IL-1β	Interleukin-1 beta
IL-6	Interleukin-6
KYN	Kynurenine
MEL	Melatonin
MyD88	Myeloid differentiation factor 88
NE	Norepinephrine
NF-κB	Nuclear factor-kappa B
Nf2	Nuclear factor erythroid 2-related factor 2
NORT	Novel object recognition experiment
OLRT	Object location recognition experiment
OFT	Open Field Test
PAT	Passive Avoidance Test
PI3K	Phosphoinositide 3-kinase
PSD95	Postsynaptic Density Protein-95
AKT	Protein kinase B
GSK3β	Glycogen synthase kinase 3 beta
QUIN	Quinolinic acid
SYN	Synaptophysin
TrkB	Tropomyosin receptor kinase B
TLR2	Toll-like receptor 2
TNFα	Tumor Necrosis Factor alpha
TRP	Tryptophan
