# Supplementary material for: Gastrodia elata, Polygonatum sibiricum, and Poria cocos as a Functional Food Formula: Cognitive Enhancement via Modulation of Hippocampal Neuroinflammation and Neuroprotection in Sleep-Restricted Mice
Source: Foods. 2025 Mar 22;14(7):1103. doi: 10.3390/foods14071103 (PMC11988919; doi:10.3390/foods14071103)
Supplement: Supplementary file 1 [file foods-14-01103-s001.zip › foods-3489790-supplementary.pdf]

Supplementary Materials

Analysis of the Chemical Composition of CGEF

This section includes a detailed description of the experimental procedures for the preparation and analysis of CGEF, as well as supplementary tables of component analysis and additional results that have been omitted from the main text for brevity. These materials provide a deeper understanding of the methods and findings presented in the manuscript.

The Compound Gastrodia elata Formula (CGEF) is composed of *Gastrodia elata* Blume (Tianma), *Polygonatum sibiricum* F. Delaroche (Huangjing), and *Poria cocos* (Schw.) Wolf (Fuling). Gastrodia elata was sourced from Chengdu, Sichuan Province, while Polygonatum sibiricum and Poria cocos were obtained from Huaihua, Hunan Province. All materials were identified by Dr. Rui-Le Pan, a researcher at the Institute of Medicinal Plant Development (IMPLAD), Chinese Academy of Medical Sciences and Peking Union Medical College, as the dried tuber of the orchid *Gastrodia elata* Blume., the dried rhizome of *Polygonatum sibiricum* F. Delaroche, and the dried sclerotium of *Poria cocos* (Schw.) Wolf from the family Polyporaceae. To elucidate the chemical foundation of CGEF, the major components of the formula were analyzed using liquid chromatography-mass spectrometry (LC-MS), providing the chemical basis for understanding the pharmacological effects of CGEF in improving cognitive impairments.

1 Materials

1.1 Reagents

Chromatographic-grade formic acid (Batch No: 2023041701) and analytical-grade methanol (Batch No: 2022092002) were purchased from Kolon Chemical Co., Ltd. (Chengdu, China); chromatographic-grade acetonitrile (Batch No: F22M8G206) was provided by Thermo Fisher

Scientific; distilled water (Batch No: 20231016) was supplied by Watsons Co., Ltd. (Guangzhou, China).

1.2 Instruments

Ultra-High Performance Liquid Chromatograph (UHPLC): Agilent 1290 UHPLC, Agilent Technologies, Inc. High-Resolution Mass Spectrometer: Agilent 6530 LC/Q-TOF, Agilent Technologies, Inc. Ultra-Pure Water System for Trace Elements: WP-Z-UV, Waterle Water Treatment Equipment Co., Ltd. (Sichuan, China). Ultrasonic Cleaner: BK-360B, Barke Ultrasonic Technology Co., Ltd. (Jinan, China)

2 Methods

2.1 Preparation of the Drug

The CGEF decoction was prepared using a water extraction method. Gastrodia elata (Tianma), Polygonatum sibiricum (Huangjing), and Poria cocos (Fuling) dried sclerotia were weighed according to a 1:1:1 (weight ratio) and immersed in water for 30 minutes. Subsequently, the mixture was extracted three times with water, with each extraction lasting 1 hour and using a 10-fold volume of water for each extraction. The three extracts were then combined, filtered through a 100-mesh sieve, and concentrated using a water bath to a final concentration of 1 g/mL of crude drug. The concentrated solution was stored at -20°C for further use. The compound formulation used in this study is covered by a patent application in China (patent application number: 202510080606.9).

2.2 Chromatographic Conditions

The chromatographic analysis was conducted under the following conditions: a Waters

ACQUITY UPLC HSS T3 column (2.1×100 mm, 1.8 μm) was used, with the mobile phase consisting of 0.1% formic acid aqueous solution as the aqueous phase and acetonitrile as the organic phase. The flow rate was set at 0.3 mL/min, the column temperature was maintained at 35°C, and the injection volume was 0.5 μL.

2.3 Mass Spectrometry Conditions

The mass spectrometric analysis was performed using an Electrospray Ionization (ESI) source in both positive and negative ion modes. The conditions were as follows: spray voltage was set to 4 kV for positive mode and 3.5 kV for negative mode. The sheath gas temperature was 350°C, with a sheath gas flow rate of 11 L/min. The drying gas flow rate was 5 L/min, and the drying gas temperature was 300°C. The scan mode used was full scan with a scan range of m/z 100 to 1700. The cone voltage was set at 100 V, and the collision energy gradients were applied at 10, 20, 40, and 60 eV.

2.4 Data Processing

The raw data collected was imported into the Qualitative Analysis 10.0 software. Using its guided setup and method templates, an unknown compound identification workflow was established to perform peak extraction on the raw data. The characteristic peaks in the samples were analyzed, and possible molecular formulas were deduced by fitting the molecular ion chromatographic peaks and isotopic peaks. Secondary fragment ions were matched with the PCDL secondary database, and results with mass deviation greater than 7.5 ppm or matching scores lower than 80 were filtered out. The secondary fragment results were also matched using the online database SIRIUS 5.8.5, selecting compounds with a match score greater than 80 and a FingerID below 50. Additionally, the Rhizoma

Polygonati Plant Composition Database, Poria cocos Plant Composition Database, and Gastrodia elata Plant Composition Database were used for primary matching of characteristic peaks that did not have secondary matches. The final results were obtained by combining the matching results from the three databases.

3 Results

The total ion chromatogram obtained from the experiment is shown below. From top to bottom, the chromatogram includes the positive ion mode, negative ion mode, and the ultraviolet absorption spectrum at a wavelength of 254 nm. Based on the database matching information, a total of 28 characteristic peaks from Gastrodia elata, Polygonatum sibiricum, and Poria cocos were identified in the sample, with the specific chemical substances represented by these peaks listed in the table. The sample detected active components such as gastrodin, p-hydroxybenzyl alcohol, and balioside compounds from Gastrodia elata, as well as various saponins, monosaccharides, and polysaccharides from Polygonatum sibiricum and Poria cocos. Some compounds, such as Polygonatum alkaloid and n-butyl-β-D-fructofuranoside, matched successfully in the Polygonatum plant database, indicating the presence of active components from Polygonatum in the sample. Additionally, chemical components like L-isoleucine and N-benzyloxycarbonyl-DL-leucine matched successfully in the Poria cocos plant database, confirming the presence of active components from Poria cocos in the sample.

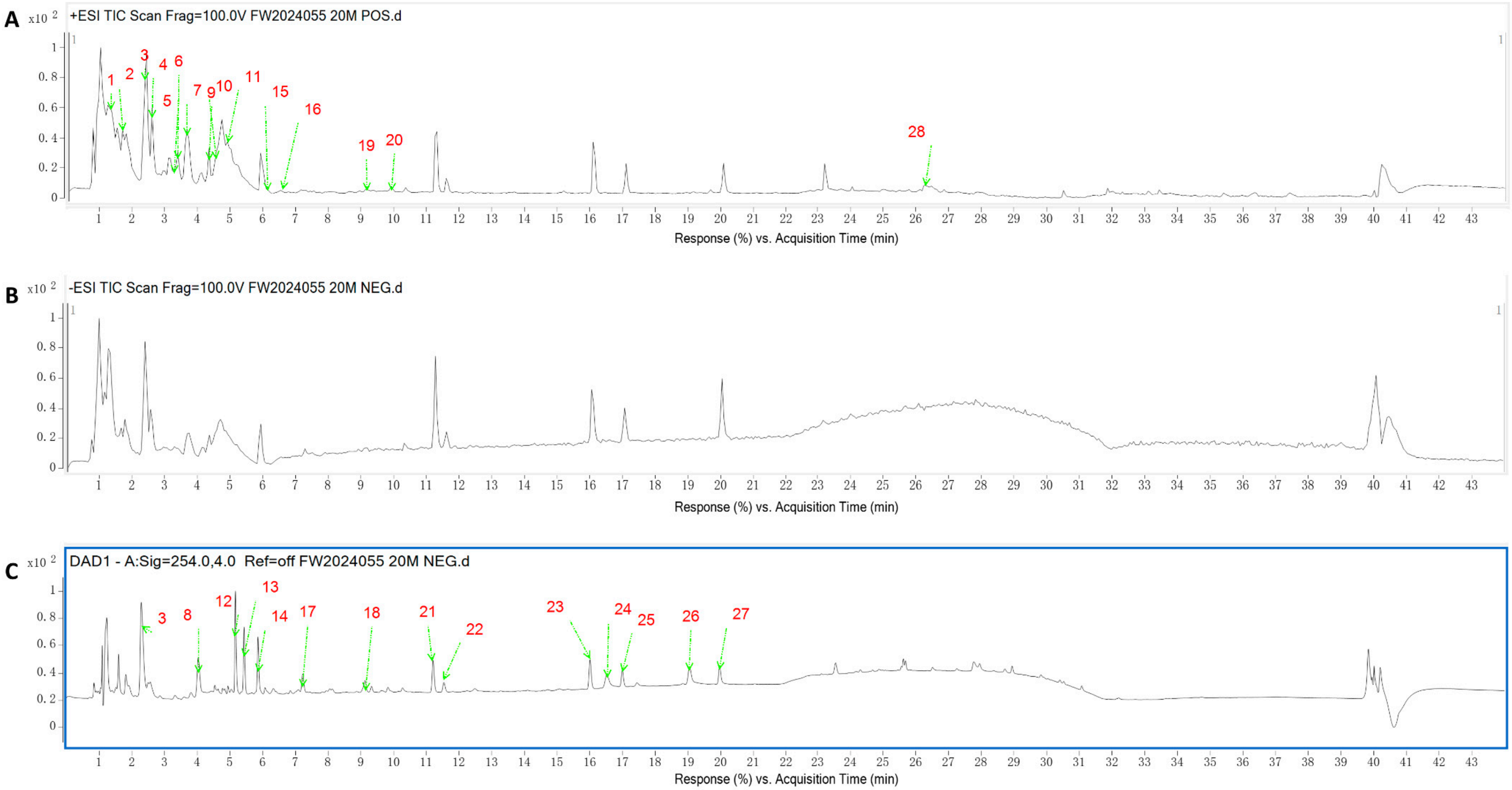

Figure S1. Identification of compounds in CGEF by LCMS. (A) Total ion chromatogram (TIC) of CGEF in positive ion mode; (B) Total ion chromatogram (TIC) of CGEF in negative ion mode; (C) UV absorbance spectrum of CGEF at 254 nm.

Table S1: Compound identification information of CGEF by LCMS.

No.	tR (min)	Molecular Formula	Theoretical Value (m/z)	Positive Mode			Negative Mode			Secondary Fragments	Identification Result	Remarks
				Experimental Value (Parent Ion)	Error (ppm)	Score	Experimental Value (Parent Ion)	Error (ppm)	Score			
1	1.279	C12H22O11	342.1162	365.1058	1.19	97.99	341.1113<br>377.0859<br>387.1142	1.24	98.19	(+)203,185,169,145<br>(-)341,179,161,143,119	Trehalose<br>Sucrose<br>Maltose	SIRIUS Online Database
2	1.673	C18H32O16	504.169	527.1589	0.36	90.6	539.1393<br>549.1675	1.73	84.19	(+)437,365,347,275,203,185<br>(-)503,341,323,179,143,119	Mannitol<br>Melezitose	PCDL Secondary Database
3	2.405	C6H8O7	192.027	193.0335<br>215.0165	-1.67	99.11	191.0213	2.59	97.22	(+)172,169,133,116<br>(-)173,160,147,129,111	Citric Acid<br>Isocitric Acid	SIRIUS Online Database
4	2.573	C5H7NO3	129.0426	130.0496<br>152.0316	-3.47	82.13	128.0345	0.92	97.05	(+)124,118,112<br>(-)122,117,112	L-Glutamic Acid	Rhizoma Polygonati Plant Database
5	3.233	C30H52O26	828.2747	851.2613	-2.79	81.06	827.2669<br>863.2358	-1.06	89.48	(+)689,527,347,185	Maltotriose	PCDL Secondary Database
6	3.360	C6H13NO2	131.0946	132.1015	-2.65	86.93	-	-	-	(+)123,119,116,114,102	L-Isoleucine	Poria cocos Plant Database
7	3.642	C24H42O21	666.2219	689.2120	1.03	92.59	665.2145<br>701.1897<br>711.2184	-2.32	96.09	(+)527,509,365,347,203,185<br>(-)485,383,341,323,179,161	Nigerose	PCDL Secondary Database

8	4.033	C8H8O	121.0648	121.0641<br>138.0909	-4.06	86.36	-	-	-	(+)119,112,104,103,100	m-Ethylphenol<br>p-Hydroxy Styrene	SIRIUS Online Database
9	4.316	C24H42O21	666.2219	689.2112	-1.49	82.57	665.2150<br>701.1913<br>711.2185	-1.29	92.78	(+)527,509,365,347,275,185<br>(-)485,341,323,221,179,161	Nigerose	PCDL Secondary Database
10	4.542	C36H62O31	990.3275	1013.3175	-0.91	95.04	989.3186<br>1025.2905	-1.04	77.35	(+)851,689,509,347,185<br>(-)827,665,545,503,383,341,179	Maltopentaose	PCDL Secondary Database
11	4.880	C42H72O36	1152.3803	1175.3697	-3.51		1151.3714<br>1187.3462	-2.98	88.21	(+)1013,851,671,509,347<br>(-)989,827,665,545,341,179	Maltosahexaose	PCDL Secondary Database
12	5.166	C10H13N5O4	267.0968	268.1039	-0.54	81.52	302.0659<br>312.0959	-	-	(+)136,119<br>(-)256,188,173,166,134	Adenosine	PCDL Secondary Database
13	5.432	C10H13N5O5	283.0917	284.0988<br>306.0800	-2.85	89.81	282.0860<br>318.0771	-	-	(+)152,135,110	Guanosine	PCDL Secondary Database
14	5.860	C13H18O7	286.1053	304.1393<br>309.0946<br>595.2003	0.33	92.46	321.0749<br>331.1039	1.66	97.9	(+)244,180,107<br>(-)285,207,161,133,105	Gastrodin	PCDL Secondary Database
		C9H8O2	148.0524	166.0859	-3.3	93.33	-	-	-	(+)149,131,120,103	Trans-Cinnamic Acid	Rhizoma Polygonati Plant Database
		C9H11NO2	165.079		-2.96	93.42	-	-	-		Polygonatum Alkaloid A	Rhizoma Polygonati Plant Database
15	6.118	C10H20O6	236.126	254.1605	2.47	95.07	-	-	-	(+)237,195,167,140,125	n-Butyl-β-D-	Rhizoma

											fructofuranoside<br>n-Butyl-O-β-D-fructopyranoside	Polygonati Plant Database
16	6.587	C10H16O8	264.0845	282.119	2.69	94.67	-	-	-	(+)241,220,199,136	Astilbin	Rhizoma Polygonati Plant Database
17	7.226	C7H8O2	124.0524	107.0487<br>125.9859	0.36	85.31	-	-	-	(+)107	p-Hydroxybenzyl Alcohol	PCDL Secondary Database
18	9.133	ion current absence										
19	9.153	C11H12N2O2	204.0899	205.0963	-3.96	94.17	-	-	-	(+)188,170,159,146,132,125,118	L-Tryptophan	Rhizoma Polygonati Plant Database
20	9.884	C11H15N5O3S	297.0896	298.0968	0.24	99.5	-	-	-	(+)163,136,119	Methylthioadenosine	Rhizoma Polygonati Plant Database
21	11.216	C19H24O13	460.1217	478.1558<br>483.1112	0.49	79.59	459.1159	1.71	97.82	(+)437,409,299,201,107<br>(-)397,173,129,111	Balisenoside E	SIRIUS Online Database
21	11.545	C17H23N3O7S	413.1257	414.1331<br>436.1131	-0.96	82.08	412.1201	3.86	88.12	(+)339,308,285,233,215,179,162<br>(-)306,288,272,254,210,179,160,143,128	L-γ-Glutamyl-S-[(4-hydroxyphenyl)methyl]-L-cysteinylglycine	PCDL Secondary Database
22	15.336	C14H19NO4	265.1314	288.1216	4.15	90.46	-	-	-	(+)255,203,182,164,136,107	N-Benzyloxycarbonyl-DL-leucine	Poria cocos Plant Database
23	16.016	C32H40O19	728.2164	746.2515<br>751.2067<br>767.1790	-0.34	95.88	727.2090	0.57	95.39	(+)567,405,299,213,107<br>(-)459,423,387,379,217,161,129,111	Balisenoside C<br>Balisenoside B	PCDL Secondary Database

24	16.538	ion current absence										
25	17.005	C32H40O19	728.2164	746.2513<br>751.2063<br>767.1777	-2.3	97.35	727.2083	-0.63	94.24	(+)567,405,299,213,107<br>(-)441,423,397,379,369,217,161,129,111	Balisenoside C<br>Balisenoside B	PCDL Secondary Database
26	19.065	ion current absence										
27	19.984	C45H56O25	996.3111	1014.3449<br>1019.3017<br>1035.2740	-2.04	91.11	995.3043<br>1031.2796	-0.3	97.85	(+)767,535,461,405,299,213<br>(-)727,688,485,423,397,369,161	Balisenoside	PCDL Secondary Database
28	26.241*	C16H32O2	256.2402	274.2736	-2.23	98.09	-	-	-	(+)268,197,164,136,121	Palmitic acid	Rhizoma Polygonati Plant Database

NOTE:

For entries marked with PCDL Secondary Library or SIRIUS Online Database, the results are based on secondary fragment matching.

In the positive mode TIC (Total Ion Chromatogram), the peak at 23.149 minutes with a molecular weight of 678 was verified using a blank solvent and is likely due to instrument residue, not a compound originally present in the sample. In the UV chromatogram at 254 nm, the peaks at 23.533 min, 25.607 min, and 27.757 min were analyzed and showed no significant ion signals, making it impossible to deduce the molecular weight and formula, so these peaks are not marked in the table and figures.
